# Improved risk scoring systems for colorectal cancer screening in Shanghai, China

**DOI:** 10.1002/cam4.4576

**Published:** 2022-03-11

**Authors:** Wei‐Miao Wu, Kai Gu, Yi‐Hui Yang, Ping‐Ping Bao, Yang‐Ming Gong, Yan Shi, Wang‐Hong Xu, Chen Fu

**Affiliations:** ^1^ Global Health Institute School of Public Health, Fudan University Shanghai China; ^2^ Shanghai Municipal Center for Disease Control & Prevention Shanghai China

**Keywords:** colorectal cancer, data mining, risk model, risk score, screening

## Abstract

**Background:**

An optimal risk‐scoring system enables more targeted offers for colonoscopy in colorectal cancer (CRC) screening. This analysis aims to develop and validate scoring systems using parametric and non‐parametric methods for average‐risk populations.

**Methods:**

Screening data of 807,695 subjects and 2806 detected cases in the first‐round CRC screening program in Shanghai were used to develop risk‐predictive models and scoring systems using logistic‐regression (LR) and artificial‐neural‐network (ANN) methods. Performance of established scoring systems was evaluated using area under the receiver operating characteristic curve (AUC), calibration, sensitivity, specificity, number of high‐risk individuals and potential detection rates of CRC.

**Results:**

Age, sex, CRC in first‐degree relatives, chronic diarrhoea, mucus or bloody stool, history of any cancer and faecal‐immunochemical‐test (FIT) results were identified as predictors for the presence of CRC. The AUC of LR‐based system was 0.642 when using risk factors only in derivation set, and increased to 0.774 by further incorporating one‐sample FIT results, and to 0.808 by including two‐sample FIT results, while those for ANN‐based systems were 0.639, 0.763 and 0.805, respectively. Better calibrations were observed for the LR‐based systems than the ANN‐based ones. Compared with the currently used initial tests, parallel use of FIT with LR‐based systems resulted in improved specificities, less demands for colonoscopy and higher detection rates of CRC, while parallel use of FIT with ANN‐based systems had higher sensitivities; incorporating FIT in the scoring systems further increased specificities, decreased colonoscopy demands and improved detection rates of CRC.

**Conclusions:**

Our results indicate the potentials of LR‐based scoring systems incorporating one‐ or two‐sample FIT results for CRC mass screening. External validation is warranted for scaling‐up implementation in the Chinese population.

## Abstract 2

The established scoring systems derived from the logistic regression (LR) models, incorporating one‐ or two‐sample faecal immunochemical test (FIT) results as a predictor, have the potential to triage high‐risk individuals for colonoscopy in mass screening of colorectal cancer (CRC). More importantly, the cut‐off points of the scoring systems can be adjusted flexibly, facilitating the choices of cut‐off values for populations with abundant or limited resources.

## 1 BACKGROUND

Colonoscopy has been widely used in colorectal cancer (CRC) screening as the reference standard to detect precancerous lesions and early‐stage cancers.^1^ The invasiveness nature and high cost of colonoscopy, as well as the constraints on capacity, however, impose restrictions on its full utilization in mass screening,^2^ particularly in countries or areas with low incidence of CRC and limited healthcare resources. A triage screening strategy, usually a colonoscopy examination following a positive result of a faecal occult blood test (FOBT), has been suggested to reduce colonoscopy requirements by around 30%,^3^, ^4^ and demonstrates huge potential in reducing the disease burden of CRC in numerous randomized trials and observational studies.^5^ However, FOBT could merely detect bleeding lesions. Therefore, multiple risk scoring systems have been developed to be jointly used with FOBT to identify high‐risk individuals with or without bleeding for further colonoscopy.^6^

Risk stratification has been widely used alone or parallel with FOBT in China, but was found leading to low adherence to colonoscopy follow‐up among those who should take.^7^, ^8^, ^9^, ^10^ The low adherence was found to be associated with the low specificity of the initial tests.^10^ In Jiashan County, Zhejiang Province of China, a risk‐stratified system was parallel used with FOBT as the initial screening tests for CRC in an average‐risk population and achieved a specificity of 81.37%.^11^ Of the high‐risk individuals identified, however, only 55.3% attended subsequent colonoscopy examination in Hangzhou City.^8^ The parallel tests achieved an adherence to colonoscopy as low as 39.8% in high‐risk subjects in Shanghai,^12^ and only 24.9% among those living in Pudong New Area of the city.^10^ In the population‐based Cancer Screening Program in Urban China (CanSPUC), the revised Harvard Risk Index was used to identify high‐risk individuals of CRC, among whom only 14.0% attended colonoscopy follow‐up.^9^ These findings cast a doubt on the suitability of the risk‐stratified systems in the Chinese population and suggest a crucial need for an optimal one.

We previously established a scoring system using the parametric logistic regression (LR) method to integrate factors of age, sex, chronic diarrhoea, mucus or bloody stool, colorectal polyps, serious unhappy life events and family history of CRC in first‐degree relatives in the residents of Pudong New Area of Shanghai.^10^ Although a score of “‐5” was assigned to the subjects with prior polyps versus those without, probably due to instant polypectomy, the scoring system still outperformed the pre‐defined risk stratification in detecting CRC. The improved performance may be partly explained by the changing etiologic spectrum of CRC and altered exposures to risk factors due to social transformation, indicating the importance to develop population‐specific real‐time risk‐assessment tools for more targeted colonoscopy screening. In this previous study, however, we did not calculate standardized risk scores to ensure reliability and comparability of scores for risk factors.^13^ We did not apply non‐parametric methods, either, which performed better than parametric approaches in the context of a large sample size and fitting non‐linear relationships.^14^

In this study, we used the data collected in the first‐round CRC screening program in Shanghai to develop and validate risk scoring systems using parametric and non‐parametric methods. Our findings may help to update the currently used risk‐assessment tool, facilitate triage of individuals for colonoscopy and improve the efficiency of the CRC screening in Shanghai.

## 2 METHODS

This large‐scale cross‐sectional study was reported according to the Strengthening the Reporting of Observational Studies in Epidemiology (STROBE)^15^ and the Transparent Reporting of a multivariable prediction model for Individual Prognosis Or Diagnosis (TRIPOD) guidelines.^16^

### 2.1 Study population

The Shanghai CRC screening program is an ongoing cascade‐screening program initiated in 2013. The program adopted a similar two‐step screening strategy used in Jiashan County,^7^, ^8^ with the parallel use of questionnaire‐based risk stratification and two‐sample qualitative faecal immunochemical test (FIT) for initial screening, followed by colonoscopy screening for participants with positive results in the initial screening.^10^, ^12^ Study population, screening protocol and data management of the study have been reported previously.^12^ Briefly, any residents aged 50–74 years old and having no prior CRC were eligible for the Shanghai CRC screening program, and the volunteers were consecutively enrolled to participate in the first round of the program in 2013. Duplicate records due to repeated participation in the program were excluded from this analysis.

### 2.2 Data collection

All participants were asked to provide sociodemographic information including date of birth, sex, educational level, marital status, occupation, residence area and to answer if they had the following events for risk stratification: (1) history of any cancer; (2) colorectal polyps; (3) CRC in first‐degree relatives; (4) chronic constipation; (5) chronic diarrhoea; (6) mucus or bloody stool; (7) serious unhappy life events such as loss of family member; (8) chronic appendicitis or appendectomy; (9) chronic cholecystitis or cholecystectomy. Participants were regarded as high‐risk if they had one of the first three events and/or at least two of the subsequent six events, similar to the risk assessment system used in the Jiashan County.^7^, ^8^

All participants were instructed to collect two faeces samples with an interval of seven days. Each sample was collected in a tube, containing about 5 mL moist stool content, and was required to return to local hospitals within 48 hours. The FIT results were read in five minutes of testing using colloidal gold assay, with a positivity threshold of 100 ng Hb/mL. The positive subjects in any FIT or in risk assessment were invited to attend colonoscopy follow‐up.

Considering the approximately 2‐year latent period of CRC,^17^ we defined the already‐present CRC at the time of screening as those diagnosed within 2 years of screening. The screen‐detected cancers were obtained from the program reporting system, and the interval or missed cancers were supplemented through conducting record‐linkage with the Shanghai Cancer Registry using unique ID number.

### 2.3 Statistical analysis

All participants completing the initial screening tests were included in this analysis. Stratified sampling was conducted to randomly select 60% of the study subjects as the derivation set (*n* = 484,321). The remaining 40% subjects were used as a validation set (*n* = 322,880). The strata were created using the outcome of CRC in STRATA options of SAS PROC SURVEYSELECT, ensuring a random and equal distribution of CRC cases in both sets.

Prior to the model development, univariable analyses and multivariable LR analyses were performed in all participants to select possible predictors for CRC from the variables collected. The LR algorithm is shown in formula $\left(1\right)$, where p is the probability of CRC case; 1−p is the probability of non‐CRC case; $\beta_{0},\beta_{1},\beta_{2},\ldots ,\beta_{k}\;$are the intercept and regression coefficients of predictors. The most important advantages of LR algorithm are the simplicity and interpretability of the model. To correct *p* values for multiple comparisons in the univariable and multivariable analyses, the false discovery rates (FDR) were calculated.^18^ Statistically significant variables (*p* values after FDR <0.05) in the univariable analyses were included in the backward elimination LR model. Those remaining significant in the multivariable analysis were used to develop the final models. The coefficient for each model predictor was transferred into a point value, with each point equivalent to the increase in the risk of CRC associated with 5‐year increase in age (i.e. the coefficient of age multiplied by 5).^13^ Then the risk score was created for each subject by summing up the point values of all predictors in the final model.

(1) $$\text{logit}\left(p\right)=\ln\left(\frac{p}{1-p}\right)=\beta_{0}+\beta_{1}*X_{1}+\beta_{2}*X_{2}+\ldots +\beta_{k}*X_{k}\;$$

Artificial neural network (ANN) method, a “black‐box” method with solid theoretical and statistical foundation, has advantages that no assumptions as to the underlying functional form between predictive variables and the outcome are required and is capable to fit both linear or non‐linear relationship between variables.^14^ The performance of ANN has been suggested superior to LR method in risk prediction of CRC,^19^ and an ANN model using the multi‐layer perceptron (MLP) was also constructed based on the significant factors in multivariable analysis. The ANN architecture based on the MLP method is organized into three layers: an input layer (predictor variables); an output layer (outcome variable); and a hidden layer (latent variables connecting predictors and outcome). All data were normalized to a value between 0 and 1 to reduce the influence of outliers and facilitate network learning using formula$\;\left(2\right)$,^20^ in which $X_{i},X_{n},X_{\min},X_{\max}\;$are the observed data, normalized data, minimum and maximum observed data, respectively. Each weighted connection reflects the strength of relationship between variables, which can be converted into risk scores according to formula $\left(3\right)$, where p is the number of predictor variables; q is the number of hidden units; $u_{\mathrm{jk}}\;$is calculated as the absolute value of weight between predictor variable k and hidden unit jdivided by total absolute values of weights pointing to hidden unit j; $v_{j}\;$is calculated as the absolute value of weight between hidden unit j and the outcome divided by total absolute values of weights pointing to the outcome; $C_{k}\;$represents the contribution of predictor variable k on the outcome; the sum of contributions of all predictor variables is 100%.^21^

(2) $$X_{n}=\frac{X_{i}-X_{\min}}{X_{\max}-X_{\min}}\;$$

(3) $$C_{k}=\sum_{j=1}^{q}v_{j}u_{\mathrm{jk}},k=1,\ldots ,p\;$$

Model discrimination and calibration were measured using the area under the receiver operating characteristic curve (AUC) and Hosmer–Lemeshow goodness‐of‐fit test.^22^ The DeLong test was applied to compare AUCs of the LR‐based and the ANN‐based scoring systems.^23^ We further calculated net reclassification improvement (NRI) and integrated discrimination improvement (IDI) between the two scoring systems.^24^ A significant positive NRI or IDI value suggests a more accurate prediction for the presence of CRC for the assessed scoring system compared to the reference one, while a negative NRI or IDI value indicates less accurate prediction.

The cut‐off points for the scoring systems were determined based on the Youden index and used to collapse risk scores of the systems into “low‐risk” and “high‐risk” categories. For the scoring systems incorporating risk factors only, the cut‐off points were also explored according to the number of high‐risk subjects identified. The scores identifying a comparable number of high‐risk subjects with the pre‐defined risk stratification were used as cut‐off points. Initial screening methods were assumed as parallel use of risk assessment with FIT results or as the scoring systems incorporating risk factors and FIT results. An optimal initial screening method was selected from these assumed methods by comparing their sensitivities, specificities, the number of high‐risk individuals identified and the potential detection rates of CRC.

The performance of models, scoring systems and assumed initial screening tests created in the derivation set were further tested in the split‐sample validation set. All statistical analyses were performed in SAS 9.4 (SAS Institute) and R (version 4.0.2). Two‐sided *p* values <0.05 were considered statistically significant.

## 3 RESULTS

### 3.1 Participant characteristics and selected risk predictors for CRC

A total of 826,445 eligible subjects were enrolled in the study, and 807,695 (97.7%) subjects completed both a questionnaire for risk‐stratification and two‐sample FITs. Demographic characteristics and screening results of the participants are presented in Table 1. The CRC cases were more likely to be men and were older at screening. A significant difference was also observed between CRC cases and non‐cases on educational level, occupation, the nine factors for risk stratification, and FIT results (*p* < 0.05, FDR <0.05).

**TABLE 1:** Characteristics and screening results of participants in the Shanghai colorectal cancer screening program

Variables	CRC cases (*n* = 2806)	Non‐cases (*n* = 804,889)	*p* values^a^	FDR^b^
Demographic characteristics				
Age at screening (years), mean (SD)	64.2 (5.7)	61.9 (6.0)	<0.001	<0.001
Sex, n (%)			<0.001	<0.001
Men	1487 (53.0)	314785 (39.1)		
Women	1319 (47.0)	490104 (60.9)		
Educational level, n (%)			0.001	0.001
No formal education	224 (8.0)	63332 (7.9)		
Primary school	760 (27.1)	230400 (28.6)		
Middle school	1498 (53.4)	436897 (54.3)		
High school	319 (11.4)	72666 (9.0)		
College or above	5 (0.2)	1594 (0.2)		
Marital status, n (%)			0.050	0.053
Married	2522 (89.9)	731574 (90.9)		
Unmarried	88 (3.1)	21419 (2.7)		
Divorced	28 (1.0)	8675 (1.1)		
Widowed	145 (5.2)	34573 (4.3)		
Unknown	23 (0.8)	8648 (1.1)		
Occupation, n (%)			0.007	0.009
Office workers	217 (7.7)	53517 (6.7)		
Enterprise workers	1190 (42.4)	324926 (40.4)		
Farmers	768 (27.4)	243836 (30.3)		
Self‐employed	64 (2.3)	19439 (2.4)		
Unemployed	119 (4.2)	33301 (4.1)		
Others	448 (16.0)	129870 (16.1)		
Resident areas, n (%)			0.175	0.175
Downtown	1026 (36.6)	284435 (35.3)		
Suburb	1780 (63.4)	520454 (64.7)		
Factors for risk stratification, *n* (%)				
Chronic diarrhoea	252 (9.0)	43204 (5.4)	<0.001	<0.001
Chronic constipation	228 (8.1)	54980 (6.8)	0.007	0.009
Mucus or bloody stool	167 (6.0)	17186 (2.1)	<0.001	<0.001
Chronic appendicitis/appendectomy	310 (11.1)	79242 (9.9)	0.033	0.037
Chronic cholecystitis/cholecystectomy	298 (10.6)	74440 (9.3)	0.012	0.015
Serious unhappy life events	86 (3.1)	17991 (2.2)	0.003	0.005
Colorectal polyps	65 (2.3)	11468 (1.4)	<0.001	<0.001
Diagnosis of any cancer	97 (3.5)	16238 (2.0)	<0.001	<0.001
CRC in first‐degree relatives	166 (5.9)	25174 (3.1)	<0.001	<0.001
Stratified as high risk	566 (20.2)	91196 (11.3)	<0.001	<0.001
Qualitative FIT positive, *n* (%)				
One‐sample	1450 (51.7)	66980 (8.3)	<0.001	<0.001
Two‐sample	1821 (64.9)	103045 (12.8)	<0.001	<0.001

Abbreviations: CRC, colorectal cancer; FDR, false discovery rate; FIT, faecal immunochemical test.

^**a**^ *p* values for *t*‐tests or chi‐square tests.

^**b**^ *p* values after FDR correction for multiple comparisons.

All significant risk factors in the univariable analyses were included in the multivariable LR model. As shown in Table S1, age at screening, sex, chronic diarrhoea, mucus or bloody stool, diagnosis of any cancer and CRC in first‐degree relatives remained significant and were identified as predictor variables for the presence of CRC. Further incorporating the results of one‐ or two‐sample FIT as an independent variable into the models did not nullify the significant associations of these factors with the presence of CRC.

### 3.2 Development and validation of predictive models

We established predictive models based on the identified predictors using LR and ANN approaches, and the ANN architectures are shown in Figures S1–S3. A total of 494 records were excluded from modelling analyses due to missing values for any one of the identified predictors. Table 2 presents the AUCs of multiple risk‐predictive models in the derivation and validation sets. The established LR and ANN algorithms using risk factors were comparable in discriminatory ability for CRC, with an AUC of 0.648 (95% CI: 0.634–0.661) for LR model and 0.651 (95% CI: 0.638–0.664) for ANN model in the derivation set (*p*_heterogeneity_ = 0.018). The AUCs increased to 0.777 (95% CI: 0.764–0.790) for the LR model and 0.779 (95% CI: 0.766–0.791) for the ANN model by further including the results of one‐sample FIT (*p*_heterogeneity_ = 0.075), and reached 0.809 (95% CI: 0.798–0.821) for LR model and 0.811 (95% CI: 0.800–0.823) for ANN model by incorporating the results of two‐sample FITs (*p*_heterogeneity_ = 0.083). The performance of the models in the validation set was very close to those in the derivation set. However, only LR models showed good calibrations (*p* > 0.05).

**TABLE 2:** Discrimination and calibration of risk predictive models for colorectal cancer in the derivation and validation sets

	Derivation set (*n* = 484,321)		Validation set (*n* = 322,880)	
	AUC (95% CI)	*p* values^a^	AUC (95% CI)	*p* values^a^
Incorporating risk factors only				
LR model	0.648 (0.634–0.661)	0.991	0.645 (0.630–0.661)	0.186
ANN model	0.651 (0.638–0.664)	<0.001	0.647 (0.632–0.663)	<0.001
Incorporating risk factors and one‐sample FIT results				
LR model	0.777 (0.764–0.790)	0.800	0.786 (0.771–0.801)	0.374
ANN model	0.779 (0.766–0.791)	<0.001	0.787 (0.772–0.802)	<0.001
Incorporating risk factors and two‐sample FIT results				
LR model	0.809 (0.798–0.821)	0.503	0.811 (0.797–0.825)	0.891
ANN model	0.811 (0.800–0.823)	<0.001	0.813 (0.799–0.826)	<0.001

Abbreviations: ANN, artificial neural network; AUC, area under the receiver operating characteristic curve; CI, confidence interval; FIT, faecal immunochemical test; LR, logistic regression.

^**a**^ *p* values for calibration based on the Hosmer–Lemeshow goodness‐of‐fit tests.

As shown in Table 3, of the risk factors, symptom of mucus or bloody stool contributed most to the risk prediction in LR model (regression coefficient: 0.91, 95% CI: 0.71–1.12), followed by CRC in first‐degree relatives, diagnosis of any cancer, sex, symptom of chronic diarrhoea and each year increase in age. Further including FIT results in the model demonstrated that FIT results contributed most to the risk prediction, with the regression coefficient as high as 2.39 (95% CI: 2.30–2.49) for the one‐sample FIT results and 2.48 (95% CI: 2.37–2.58) for the two‐sample results.

**TABLE 3:** Scoring algorithm to calculate the point values in the derivation set

Variable	Reference value (W_ij_)	Risk factors only			Incorporating one‐sample FIT results			Incorporating two‐sample FIT results		
		β (95% CI)^a^	LR score^b^	ANN score^c^	β (95% CI)^a^	LR score^b^	ANN score^d^	β (95% CI)^a^	LR score^b^	ANN score^e^
Age at screening		0.06 (0.05–0.07)			0.06 (0.05–0.07)			0.06 (0.05–0.06)		
Age group										
50–54	52 (W_ref_)	—	0	0	—	0	0	—	0	0
55–59	57	—	1.0	0.75	—	1.0	0.50	—	1.0	0.375
60–64	62	—	2.0	1.50	—	2.0	1.00	—	2.0	0.750
65–69	67	—	3.0	2.25	—	3.0	1.50	—	3.0	1.125
70–74	72	—	4.0	3.0	—	4.0	2.0	—	4.0	1.5
Sex										
Women	0 (W_ref_)	—	0	0	—	0	0	—	0	0
Men	1	0.46 (0.36–0.55)	2.0	2.0	0.39 (0.29–0.49)	1.0	2.0	0.39 (0.29–0.48)	1.0	1.5
Chronic diarrhoea										
Never	0 (W_ref_)	—	0	0	—	0	0	—	0	0
Ever	1	0.35 (0.17–0.55)	1.0	1.0	0.26 (0.09–0.44)	1.0	2.0	0.24 (0.06–0.42)	1.0	2.0
Mucus or bloody stool										
Never	0 (W_ref_)	—	0	0	—	0	0	—	0	0
Ever	1	0.91 (0.71–1.12)	3.0	2.0	0.74 (0.53–0.94)	2.0	2.5	0.70 (0.49–0.91)	2.0	2.0
Diagnosis of any cancer										
Never	0 (W_ref_)	—	0	0	—	0	0	—	0	0
Ever	1	0.51 (0.26–0.76)	2.0	2.0	0.49 (0.24–0.74)	2.0	2.0	0.48 (0.22–0.73)	2.0	2.5
CRC in first degree relatives										
No	0 (W_ref_)	—	0	0	—	0	0	—	0	0
Yes	1	0.57 (0.36–0.78)	2.0	4.0	0.49 (0.28–0.70)	2.0	3.5	0.47 (0.25–0.68)	2.0	2.5
Qualitative FIT										
Negative	—	—	—	—	—	0	0	—	0	0
Positive	—	—	—	—	2.39 (2.30–2.49)	8.0	6.0	2.48 (2.37–2.58)	8.0	8.0
Overall score	—	—	0–14	0–14	—	0–20	0–20	—	0–20	0–20

Abbreviations: ANN, artificial neural network; CI, confidence interval; CRC, colorectal cancer; FIT, faecal immunochemical test; LR, logistic regression.

^**a**^ β (95% CI) derived from multivariable LR model.

^**b**^ LR‐based risk score = β*(W_ij_‐W_ref_)/B, in which constant B is the number of regression unit equivalent to 1 point in the final risk score, and was calculated by multiplying the β for age (0.06) by 5 (0.06*5 = 0.30). Based on an age‐standardized method, the point values of other variables were obtained with their corresponding regression coefficients dividing by 0.30 and rounding to the nearest whole number, e.g. for the LR‐based scoring system with risk factors only, 0.46/0.30 = 2.0 for sex, 0.35/0.30 = 1.0 for chronic diarrhoea, 0.91/0.30 = 3.0 for mucus or bloody stool, 0.51/0.30 = 2.0 for prior diagnosis of any cancer, 0.57/0.30 = 2.0 for CRC in first degree relatives.

^**c**^ Computed by multiplying the total score in the LR‐based scoring system (14 scores) by the contribution of predictors on the outcome in the ANN model: age (20.5%), sex (17.0%), chronic diarrhoea (10.4%), mucus or bloody stool (11.7%), history of any cancer (13.3%) and CRC in first‐degree relatives (27.1%).

^**d**^ Computed by multiplying the total score in the LR‐based scoring system (20 scores) by the contribution of predictors on the outcome in the ANN model: age (9.9%), sex (10.7%), chronic diarrhoea (10.5%), mucus or bloody stool (12.3%), history of any cancer (10.2%), CRC in first‐degree relatives (18.4%) and one‐sample FIT (28.0%).

^**e**^ Computed by multiplying the total score in the LR‐based scoring system (20 scores) by the contribution of predictors on the outcome in the ANN model: age (7.6%), sex (7.3%), chronic diarrhoea (10.7%), mucus or bloody stool (9.7%), history of any cancer (11.8%), CRC in first‐degree relatives (13.5%) and two‐sample FIT (39.4%).

### 3.3 Development and validation of scoring systems

Table 3 also shows the scoring algorithms used to calculate point values based on the LR and ANN models in the derivation set. The scores ranged from 0 to 14 for LR models derived from risk factors, and from 0 to 20 by further incorporating one‐ or two‐sample FIT results.

The contribution of each predictor to the outcome was further estimated and scored according to its respective weight in the ANN models. Inputting risk factors into ANN model showed that 20.5%, 17.0%, 10.4%, 11.7%, 13.3% and 27.1% of the outcome were accounted by age, sex, chronic diarrhoea, mucus or bloody stool, diagnosis of any cancer and CRC in first‐degree relatives, respectively. Further incorporating FIT results into the ANN models resulted in the largest contribution of one‐sample (28.0%) or two‐sample FIT results (39.4%) to the outcome (Tables S2–S4). The risk scores were further rescaled into 14‐point or 20‐point scoring systems to be compared with the LR‐based scoring systems directly. It is of note that the ANN‐based systems assigned a higher score to “CRC in first‐degree relatives” than the LR‐based systems, but a lower score to “age at screening.”

Incorporating the FIT results greatly improved discriminations of the LR‐ and the ANN‐based scoring systems. As shown in Table 4, the AUC was 0.642 (0.629–0.655) for the LR‐based system including risk factors only and increased to 0.774 (0.761–0.787) by further incorporating one‐sample FIT results and 0.808 (0.796–0.819) by including two‐sample FIT results. The AUCs for the ANN‐based scoring systems were also observed to increase from 0.639 (0.626–0.652) to 0.763 (0.751–0.776) or 0.805 (0.793–0.817) by incorporating one‐ or two‐sample FIT results.

**TABLE 4:** Discrimination and calibration of risk scoring systems for colorectal cancer in the derivation and validation sets

	Derivation set (*n* = 484,321)		Validation set (*n* = 322,880)	
	AUC (95% CI)	*p* values^a^	AUC (95% CI)	*p* values^a^
Incorporating risk factors only				
LR‐based scoring system	0.642 (0.629–0.655)	0.967	0.641 (0.626–0.657)	0.938
ANN‐based scoring system	0.639 (0.626–0.652)	0.053	0.640 (0.624–0.655)	0.109
Incorporating risk factors and one‐sample FIT results				
LR‐based scoring system	0.774 (0.761–0.787)	0.998	0.786 (0.771–0.800)	0.923
ANN‐based scoring system	0.763 (0.751–0.776)	<0.001	0.781 (0.766–0.796)	0.002
Incorporating risk factors and two‐sample FIT results				
LR‐based scoring system	0.808 (0.796–0.819)	0.871	0.811 (0.798–0.825)	0.860
ANN‐based scoring system	0.805 (0.793–0.817)	0.001	0.809 (0.795–0.823)	0.124

Abbreviations: ANN, artificial neural network; AUC, area under the receiver operating characteristic curve; CI, confidence interval; FIT, faecal immunochemical test; LR, logistic regression.

^**a**^ *p* values for calibration based on the Hosmer–Lemeshow goodness‐of‐fit tests.

The LR‐based systems performed better in predicting the presence of CRC than the ANN‐based systems, with an NRI of 0.359 (0.313–0.404) for the systems including risk factors only, 0.194 (0.147–0.241) for the systems incorporating one‐sample FIT results, and 0.173 (0.126–0.221) for the systems incorporating two‐sample FIT results. The corresponding IDIs were 0.0001 (0.0001–0.0002), 0.0009 (0.0005–0.0013) and 0.0005 (0.0003–0.0007). Better calibrations were also observed for the LR‐based compared to the ANN‐based systems.

### 3.4 Performance of assumed initial screening methods

Based on the Youden index, the scores “4”, “6” or “7” were selected as the cut‐off points for the LR‐based scoring system including risk factors only, further incorporating one‐sample FIT results, or further incorporating two‐sample FIT results, respectively, while the scores “3”, “6” or “7” were selected for the corresponding ANN‐based scoring systems. The selected cut‐off points for scoring systems including risk factors only were found to classify more than 43.7% of subjects as high‐risk individuals when parallel used with the two‐sample FITs, which obviously were not feasible in practice for cost‐effectiveness consideration. On the other hand, the score “6” for the LR‐based system and the score “5” for the ANN‐based system were observed to classify comparable number of high‐risk individuals with that of the pre‐defined risk stratification and were used as the cut‐off points to be parallel used with the FIT.

As shown in Table 5, parallel use of the LR‐based scoring system with one‐ or two‐sample FIT was found to have higher specificities, classify less subjects as high‐risk individuals and show higher detection rates of CRC, but had lower sensitivities than the initial screening tests used in the program, namely, parallel use of pre‐defined risk stratification with two‐sample FIT. Parallel use of the ANN‐based scoring system with one‐ or two‐sample FIT were comparable in specificity, number of high‐risk individuals and potential detection rates of CRC with the initial tests of the program, but achieved higher sensitivities.

**TABLE 5:** Performance of selected initial screening methods using risk factors and FIT results in the derivation and validation sets

Initial screening methods	Derivation set (*n* = 484,321)				Validation set (*n* = 322,880)			
	No. of high‐risk subjects	No. of CRC covered (%)	Sensitivity (95% CI), %	Specificity (95% CI), %	No. of high‐risk subjects	No. of CRC covered (%)	Sensitivity (95% CI), %	Specificity (95% CI), %
Incorporating risk factors only								
Pre‐defined risk stratification	54862	335 (0.61)	19.9 (18.1–21.9)	88.7 (88.6–88.8)	36835	231 (0.63)	20.6 (18.3–23.1)	88.6 (88.5–88.7)
LR‐based scoring ≥6	42836	341 (0.80)	20.3 (18.4–22.3)	91.2 (91.1–91.3)	28644	221 (0.77)	19.7 (17.5–22.2)	91.2 (91.1–91.3)
ANN‐based scoring ≥5	53782	401 (0.75)	23.8 (21.9–25.9)	88.9 (88.9–89.0)	36033	259 (0.72)	23.1 (20.7–25.6)	88.9 (88.8–89.0)
Qualitative FIT only								
One‐sample	40946	866 (2.11)	51.5 (49.1–53.9)	91.7 (91.6–91.8)	27421	581 (2.12)	51.8 (48.9–54.7)	91.7 (91.6–91.8)
Two‐sample	62830	1094 (1.74)	65.0 (62.7–67.3)	87.2 (87.1–87.3)	41949	724 (1.73)	64.6 (61.7–67.3)	87.2 (87.1–87.3)
Incorporating risk factors and one‐sample FIT								
LR‐based scoring ≥6	50339	915 (1.82)	54.4 (52.0–56.8)	89.8 (89.7–89.8)	33496	611 (1.82)	54.5 (51.6–57.4)	89.8 (89.7–89.9)
ANN‐based scoring ≥6	56287	936 (1.66)	55.7 (53.3–58.0)	88.5 (88.4–88.6)	37701	630 (1.67)	56.2 (53.2–59.0)	88.5 (88.4–88.6)
Incorporating risk factors and two‐sample FIT								
LR‐based scoring ≥7	65753	1110 (1.69)	66.0 (63.7–68.2)	86.6 (86.5–86.7)	43848	734 (1.67)	65.5 (62.7–68.2)	86.6 (86.5–86.7)
ANN‐based scoring ≥7	64344	1102 (1.71)	65.5 (63.2–67.8)	86.9 (86.8–87.0)	42950	730 (1.70)	65.1 (62.2–67.8)	86.9 (86.8–87.0)
Parallel use of scoring system with one‐sample FIT								
Pre‐defined risk stratification	89212	1018 (1.14)	60.5 (58.2–62.8)	81.7 (81.6–81.8)	59828	683 (1.14)	60.9 (58.0–63.7)	81.6 (81.5–81.8)
LR‐based scoring ≥6	79136	1001 (1.26)	59.5 (57.2–61.8)	83.8 (83.7–83.9)	52912	685 (1.29)	61.1 (58.2–63.9)	83.8 (83.6–83.9)
ANN‐based scoring ≥5	89058	1034 (1.16)	61.5 (59.1–63.8)	81.8 (81.7–81.9)	59600	708 (1.19)	63.1 (60.2–65.9)	81.7 (81.6–81.8)
Parallel use of scoring system with two‐sample FIT								
Pre‐defined risk stratification	107718	1208 (1.12)	71.8 (69.6–73.9)	77.9 (77.8–78.1)	72060	805 (1.12)	71.8 (69.1–74.4)	77.9 (77.7–78.0)
LR‐based scoring ≥6	98651	1186 (1.20)	70.5 (68.3–72.6)	79.8 (79.7–79.9)	65834	801 (1.22)	71.5 (68.7–74.0)	79.8 (79.7–79.9)
ANN‐based scoring ≥5	107995	1213 (1.12)	72.1 (69.9–74.2)	77.9 (77.8–78.0)	72138	817 (1.13)	72.8 (70.1–75.3)	77.8 (77.7–78.0)

Abbreviations: ANN, artificial neural network; CI, confidence interval; CRC, colorectal cancer; FIT, faecal immunochemical test; LR, logistic regression; No., number.

Incorporating one‐ or two‐sample FIT results in the scoring systems performing better than parallel use of these two methods, for the higher specificities, less high‐risk individuals identified for colonoscopy, and higher detection rates of CRC, albeit at the cost of compromised sensitivities.

The performance of the potential initial screening strategies in the validation set was very similar to those in the derivation set, as shown in Table 5.

### 3.5 Sensitivity analysis

To test the predictive value of the seven identified variables for the presence of CRC, we used support vector machine (SVM) and random forest (RF) approaches to verify the results based on the LR and the ANN methods. SVM is a binary classifier apt at solving problems like small sample size, non‐linear relationship and high‐dimension pattern recognition. RF is an ensemble classifier that produces multiple decision trees using a randomly selected subset of training samples and variables. The predicted result is determined by a majority of votes among trees. The importance of variables ranked by the RF approach was identical to that derived from the LR model. As shown in Figure S4, the AUCs of RF and SVM models were observed to be close to those of the LR and the ANN models in both derivation and validation sets.

We also assumed 1‐year or 1.5‐year interval between two rounds of screening to run sensitivity analysis. We identified the same variables to develop predictive models and scoring systems and observed slightly higher AUCs of the models and the derived scoring systems than those using a 2‐year interval (Figure S5). Consistently, better calibrations were observed for the LR‐based scoring systems than the ANN‐based ones.

## 4 DISCUSSION

In this study, we developed and validated multiple risk scoring systems to identify high‐risk individuals for CRC among an average‐risk population in China. The risk scoring systems derived from the LR model incorporating age at screening, sex, chronic diarrhoea, mucus or bloody stool, diagnosis of any cancer, CRC in first‐degree relatives and the FIT results perform well in the Chinese population and have potential to be used in mass screening of CRC. More importantly, the cut‐off points of the scoring systems can be adjusted flexibly, facilitating the choices of cut‐off values for populations with abundant or limited resources.

Multiple predictive models have been established to predict advanced colorectal neoplasia in various screening settings. These models incorporated several common risk factors like age, sex, CRC in first‐degree relatives, body mass index (BMI) and smoking, but differed in choices of other predictors.^25^ While some models additionally included diarrhoea, constipation, abdominal mass and other symptoms and signs,^26^ some others integrated medications and laboratory measurements,^19^ or physical activity and dietary factors.^22^, ^27^ The LR model established in this study included six easy‐to‐collect demographic and clinical factors, somewhat consistent with several previous studies,^28^, ^29^ but less than those (demographics, lifestyle factors, medical history and genetic variants) used by Jeon et al.^30^ Considering that data mining‐based risk models had higher AUC than LR models in clinical settings,^19^ we developed ANN models using the same six or seven variables but did not observe better performance in discrimination and calibration. The inconsistency of our results with previous studies may be explained by the non‐linear relationship of the predictors with CRC in clinical settings, but linear in our large‐scale data.^14^ In this study, the AUC varied between 0.648 and 0.809 for LR models and ranged from 0.651 to 0.811 for ANN models, superior to the pre‐defined risk‐stratification in the program and those (AUC: 0.53–0.63) developed by Jeon et al,^30^ and comparable to several established models (AUC: 0.62–0.77).^25^, ^31^

To facilitate model communication, we established risk scoring systems based on the developed models to identify high‐risk subjects for colonoscopy. We found that the LR‐based scoring systems incorporating one‐ or two‐sample FIT results outperformed the ANN‐based ones in discrimination (indicated by AUC, NRI and IDI), calibration, potential detection rate of CRC and specificity, all of which were comparable to those in previous studies.^31^, ^32^ It is of note that, when using a low score for cut‐off value, more participants would be identified as high‐risk individuals, but more CRC cases would be detected, which may greatly increase the cost of screening due to the incremental demand for colonoscopy. Trade‐off should be made between the effectiveness and the cost of screening when selecting the cut‐off value of scoring. When using the score “6” or “7” as the cut‐off values, the LR‐based scoring systems incorporating one‐ or two‐sample FIT results were superior to the ANN‐based ones with respect to the specificity, which has important implications for the triage screening of CRC to reduce unnecessary colonoscopy.^33^

We also found that the performance of initial tests can be further improved by incorporating FIT results into the scoring systems instead of parallel use of the scoring systems with FIT results. Stegeman et al^34^ also provided supporting evidence for incorporating FIT results as a predictor in the scoring system to select high‐risk subjects for colonoscopy. Interestingly, parallel use of the scoring systems with FIT results was more likely to improve the sensitivity, while incorporating FIT results into the scoring systems tended to increase specificity, which is exactly the concern that urgently needs to be addressed to enhance adherence to colonoscopy follow‐up, and thereby facilitating timely detection and treatment of CRC.

Compared with clinically detected CRCs, screen‐detected CRCs have been found more likely to be at early‐stage, be treated by local excision, and have longer survival time.^12^, ^35^ A great potential of the LR‐based risk scoring systems incorporating one‐ or two‐sample FIT results is highly expected in real‐world practices of CRC screening. Regardless of the choice of one‐ or two‐sample FIT, which greatly depends on the availability of medical resources as well as population adherence, the systems can be used alone as an APP or in conjunction with other screening tests (e.g., blood testing for CRC markers) to better identify high‐risk individuals for CRC. For clinicians, the systems may help to reduce the workload by decreasing unnecessary colonoscopies; for high‐risk individuals, the systems could facilitate self‐assessment of risk for CRC, and thereby adopt healthy lifestyles to lower the risk; for policymakers, the systems would be more cost‐effective and can be used to improve and update screening strategies for CRC.

This study has several strengths. First, the analysis was based on large‐scale CRC screening data, providing enough statistical power for development and validation of the predictive models and the scoring systems. Second, a standardized protocol was used in the program for data collection, guaranteeing the quality of data. Third, the predictive models and scoring systems were derived from an average‐risk population and included only several variables easy‐to‐collect, facilitating its utility in the real world. Finally, we reported the process of model development and validation according to the TRIPOD statement and created scoring systems using an age‐standardized method based on LR coefficients and using weights in the ANN model, ensuring the validity of the scoring systems.

However, there are several limitations to this study. First, as the participants of the program were enrolled voluntarily, selection bias could not be excluded. Second, all factors for risk assessment were self‐reported, which may introduce recall bias. Moreover, as the scoring systems were developed based on available variables and did not include smoking and BMI, two common predictors in other populations, our systems may not be the optimal one. In Chinese adults, however, neither smoking nor BMI was significantly associated with the presence of advanced colorectal neoplasia.^9^ Furthermore, the huge sex disparity in smoking making sex a good proxy for smoking in the population. These facts partly released our concern. Finally, we only applied the split‐sample validation in this analysis. External validation is required to verify the robustness of predictive models and scoring systems.

In conclusion, the LR‐based scoring systems incorporating one‐ or two‐sample FIT results as a predictor have the potential to triage high‐risk individuals for colonoscopy in CRC screening in Chinese adults. The scoring systems facilitate the flexible choices of cut‐off values to ensure the efficiency of the CRC screening. External validation is needed for its scaling‐up implementation in real‐life practice.

## CONFLICT OF INTEREST

The authors declare no conflict of interest.

## Funding information

This study was supported by the National Key R&D Program of China (No. 2017YFC1308800), the Health Commission of the Pudong New Area of Shanghai (No. PW2019A‐5) and the Key Technology Research for Colorectal Cancer Screening and High‐risk Population Follow‐up (No. 20DZ1100103).

## AUTHORS' CONTRIBUTIONS

Conceptualization and design: W.H.X. and C.F.; Collection and assembly of data: P.P.B., Y.M.G., K.G. and Y.S.; Data analysis and interpretation: W.M.W., Y.H.Y. and K.G.; Manuscript writing: W.M.W. and K.G.; Final approval of manuscript: All authors.

## ETHICAL APPROVAL STATEMENT

The Shanghai CRC screening program was approved by the Ethics Review Committee of the Shanghai Municipal Center for Disease Control & Prevention. Written informed consent was obtained from all study participants.

## Supporting information


Supinfo

## Data Availability

The datasets generated and/or analysed during the current study are available from the corresponding author on reasonable request.
