# Supplementary material for: Improved risk scoring systems for colorectal cancer screening in Shanghai, China
Source: Cancer Med. 2022 Mar 11;11(9):1972–83. doi: 10.1002/cam4.4576 (PMC9089226; doi:10.1002/cam4.4576)
Supplement: Supplementary file 1 — Supinfo [file CAM4-11-1972-s001.pdf]

Supplementary Materials

Supplementary Table 1. Multivariable logistic regression analysis of risk factors for colorectal cancer (n=807,201)

Variable	Without FIT				Incorporating 1-sample FIT				Incorporating 2-sample FIT			
	β	SE	OR (95%CI)	*p* value / FDR	β	SE	OR (95%CI)	*p* value / FDR	β	SE	OR (95%CI)	*p* value / FDR
Age at screening (years)	0.061	0.003	1.062 (1.056, 1.069)	< 0.001	0.056	0.003	1.058 (1.051, 1.064)	< 0.001	0.055	0.003	1.056 (1.049, 1.063)	< 0.001
Sex (male vs female)	0.456	0.038	1.578 (1.464, 1.701)	< 0.001	0.386	0.038	1.471 (1.364, 1.586)	< 0.001	0.382	0.038	1.465 (1.359, 1.580)	< 0.001
Chronic diarrhea	0.392	0.068	1.480 (1.295, 1.692)	< 0.001	0.300	0.069	1.350 (1.180, 1.544)	< 0.001	0.277	0.069	1.318 (1.153, 1.508)	< 0.001
Mucus or bloody stool	0.870	0.083	2.386 (2.029, 2.806)	< 0.001	0.683	0.083	1.980 (1.682, 2.331)	< 0.001	0.646	0.083	1.907 (1.620, 2.245)	< 0.001
Diagnosis of any cancer	0.402	0.104	1.495 (1.219, 1.834)	< 0.001	0.377	0.105	1.458 (1.187, 1.792)	< 0.001	0.367	0.105	1.444 (1.175, 1.774)	< 0.001
CRC in first-degree relatives	0.631	0.081	1.880 (1.604, 2.203)	< 0.001	0.554	0.082	1.740 (1.483, 2.041)	< 0.001	0.531	0.081	1.701 (1.450, 1.995)	< 0.001
FIT (positive vs negative)	-	-	-	-	2.395	0.038	10.972 (10.181, 11.825)	< 0.001	2.465	0.040	11.764 (10.880, 12.719)	< 0.001

CRC, colorectal cancer; FIT, faecal immunochemical test; OR, odds ratio; FDR, false discovery rate (corrected *p* value for multiple comparisons).

Supplementary Table 2. Weights from the input variables to the hidden layer neurons, and from the hidden layer neurons to the outcome variable in the artificial neural network model incorporating risk factors only

From	To	Weight	Abs (weight)	Sum	Abs (weight)/Sum	$v_j * u_{jk}$	C_k
Male	H1	-0.27174	0.27174	0.97661	0.27825	0.01161	0.170
Diagnosis of any cancer	H1	-0.26207	0.26207		0.26834	0.01120	0.133
Age at screening	H1	-0.15460	0.15460		0.15830	0.00661	0.205
Chronic diarrhea	H1	0.12497	0.12497		0.12796	0.00534	0.104
Mucus or bloody stool	H1	0.08865	0.08865		0.09077	0.00379	0.117
CRC in 1st degree relatives	H1	0.07459	0.07459		0.07637	0.00319	0.271
Age at screening	H2	0.19650	0.19650	0.63782	0.30807	0.00920	
CRC in 1st degree relatives	H2	0.16219	0.16219		0.25428	0.00759	
Male	H2	0.13984	0.13984		0.21924	0.00655	
Chronic diarrhea	H2	-0.10297	0.10297		0.16144	0.00482	
Diagnosis of any cancer	H2	0.03475	0.03475		0.05449	0.00163	
Mucus or bloody stool	H2	0.00158	0.00158		0.00248	0.00007	
Mucus or bloody stool	H3	2.01889	2.01889	8.35712	0.24158	0.03016	
CRC in 1st degree relatives	H3	1.94547	1.94547		0.23279	0.02907	
Male	H3	-1.55340	1.55340		0.18588	0.02321	
Age at screening	H3	-1.53159	1.53159		0.18327	0.02288	
Chronic diarrhea	H3	-0.67775	0.67775		0.08110	0.01013	
Diagnosis of any cancer	H3	0.63004	0.63004		0.07539	0.00941	
Age at screening	H4	1.93165	1.93165	5.05783	0.38191	0.03961	
CRC in 1st degree relatives	H4	-1.23122	1.23122		0.24343	0.02525	
Diagnosis of any cancer	H4	0.97622	0.97622		0.19301	0.02002	
Chronic diarrhea	H4	0.73138	0.73138		0.14460	0.01500	
Male	H4	0.18253	0.18253		0.03609	0.00374	
Mucus or bloody stool	H4	-0.00483	0.00483		0.00095	0.00010	
CRC in 1st degree relatives	H5	2.68647	2.68647	5.93873	0.45236	0.09853	
Age at screening	H5	1.14948	1.14948		0.19356	0.04216	
Mucus or bloody stool	H5	1.05097	1.05097		0.17697	0.03854	
Diagnosis of any cancer	H5	0.49258	0.49258		0.08294	0.01807	
Male	H5	0.47707	0.47707		0.08033	0.01750	
Chronic diarrhea	H5	0.08217	0.08217		0.01384	0.00301	
Male	H6	-2.09256	2.09256	6.76293	0.30942	0.04190	
CRC in 1st degree relatives	H6	-2.00520	2.00520		0.29650	0.04015	
Chronic diarrhea	H6	-1.01957	1.01957		0.15076	0.02041	
Age at screening	H6	-0.59480	0.59480		0.08795	0.01191	
Diagnosis of any cancer	H6	0.58950	0.58950		0.08717	0.01180	
Mucus or bloody stool	H6	0.46129	0.46129		0.06821	0.00924	
Age at screening	H7	2.46842	2.46842	8.21875	0.30034	0.03497	
Male	H7	1.82274	1.82274		0.22178	0.02582	
Mucus or bloody stool	H7	-1.18750	1.18750		0.14449	0.01682	
Chronic diarrhea	H7	0.93882	0.93882		0.11423	0.01330	
Diagnosis of any cancer	H7	0.92094	0.92094		0.11205	0.01305	
CRC in 1st degree relatives	H7	0.88033	0.88033		0.10711	0.01247	
Diagnosis of any cancer	H8	0.65124	0.65124	1.62567	0.40060	0.02459	
Chronic diarrhea	H8	-0.42257	0.42257		0.25993	0.01596	
CRC in 1st degree relatives	H8	0.20312	0.20312		0.12494	0.00767	
Mucus or bloody stool	H8	0.15098	0.15098		0.09287	0.00570	

Age at screening	H8	0.10092	0.10092		0.06208	0.00381
Male	H8	0.09685	0.09685		0.05958	0.00366
Age at screening	H9	0.92719	0.92719	2.87844	0.32212	0.00744
Male	H9	0.51864	0.51864		0.18018	0.00416
Chronic diarrhea	H9	-0.43434	0.43434		0.15089	0.00348
CRC in 1st degree relatives	H9	-0.40795	0.40795		0.14173	0.00327
Diagnosis of any cancer	H9	0.38351	0.38351		0.13324	0.00308
Mucus or bloody stool	H9	0.20681	0.20681		0.07185	0.00166
Age at screening	H10	-1.01713	1.01713	4.12186	0.24677	0.01721
CRC in 1st degree relatives	H10	0.92324	0.92324		0.22399	0.01562
Male	H10	-0.82160	0.82160		0.19933	0.01390
Diagnosis of any cancer	H10	-0.70272	0.70272		0.17049	0.01189
Chronic diarrhea	H10	0.64789	0.64789		0.15718	0.01096
Mucus or bloody stool	H10	-0.00927	0.00927		0.00225	0.00016
CRC in 1st degree relatives	H11	0.55002	0.55002	1.45946	0.37686	0.02864
Male	H11	0.35183	0.35183		0.24107	0.01832
Mucus or bloody stool	H11	0.19413	0.19413		0.13302	0.01011
Age at screening	H11	0.18169	0.18169		0.12449	0.00946
Diagnosis of any cancer	H11	-0.16039	0.16039		0.10990	0.00835
Chronic diarrhea	H11	0.02140	0.02140		0.01467	0.00111
H5	CRC	1.75004	1.75004	8.03495	0.21780	
H6	CRC	-1.08799	1.08799		0.13541	
H3	CRC	1.00322	1.00322		0.12486	
H7	CRC	-0.93545	0.93545		0.11642	
H4	CRC	0.83334	0.83334		0.10371	
H11	CRC	-0.61066	0.61066		0.07600	
H10	CRC	-0.56034	0.56034		0.06974	
H8	CRC	-0.49327	0.49327		0.06139	
H1	CRC	0.33528	0.33528		0.04173	
H2	CRC	-0.23987	0.23987		0.02985	
H9	CRC	0.18549	0.18549		0.02308	

CRC, colorectal cancer. Note: u_{jk} is calculated as the absolute value of weight between predictor variable k and hidden unit j divided by total absolute values of weights pointing to hidden unit j; v_j is calculated as the absolute value of weight between hidden unit j and the outcome divided by total absolute values of weights pointing to the outcome; C_k represents the contribution of predictor variable k on the outcome; the sum of contributions of all predictor variables is 100%.

Supplementary Table 3. Weights from the input variables to the hidden layer neurons, and from the hidden layer neurons to the outcome variable in the artificial neural network model incorporating risk factors and 1-sample FIT results

From	To	Weight	Abs (weight)	Sum	Abs (weight)/Sum	$v_j * u_{jk}$	C_k
Age at screening	H1	0.29252	0.29252	1.42158	0.20577	0.00669	0.099
Diagnosis of any cancer	H1	0.10606	0.10606		0.07461	0.00243	0.102
Positive FIT (1-sample)	H1	0.26350	0.26350		0.18536	0.00603	0.280
Chronic diarrhea	H1	-0.05941	0.05941		0.04179	0.00136	0.105
Male	H1	-0.13967	0.13967		0.09825	0.00319	0.107
Mucus or bloody stool	H1	0.17779	0.17779		0.12507	0.00407	0.123
CRC in 1st degree relatives	H1	0.38261	0.38261		0.26915	0.00875	0.184
Age at screening	H2	0.23872	0.23872	2.85752	0.08354	0.00936	
Diagnosis of any cancer	H2	0.02195	0.02195		0.00768	0.00086	
Positive FIT (1-sample)	H2	-0.38582	0.38582		0.13502	0.01512	
Chronic diarrhea	H2	-0.57296	0.57296		0.20051	0.02246	
Male	H2	-0.19409	0.19409		0.06792	0.00761	
Mucus or bloody stool	H2	-0.72617	0.72617		0.25413	0.02846	
CRC in 1st degree relatives	H2	0.71780	0.71780		0.25120	0.02813	
Age at screening	H3	0.07186	0.07186	1.94706	0.03691	0.00236	
Diagnosis of any cancer	H3	-0.18630	0.18630		0.09568	0.00613	
Positive FIT (1-sample)	H3	-0.66061	0.66061		0.33929	0.02174	
Chronic diarrhea	H3	-0.25302	0.25302		0.12995	0.00833	
Male	H3	0.31689	0.31689		0.16275	0.01043	
Mucus or bloody stool	H3	-0.00934	0.00934		0.00480	0.00031	
CRC in 1st degree relatives	H3	-0.44903	0.44903		0.23062	0.01478	
Age at screening	H4	0.49424	0.49424	2.58054	0.19153	0.01832	
Diagnosis of any cancer	H4	0.02298	0.02298		0.00891	0.00085	
Positive FIT (1-sample)	H4	0.84717	0.84717		0.32829	0.03140	
Chronic diarrhea	H4	-0.10517	0.10517		0.04075	0.00390	
Male	H4	0.33989	0.33989		0.13171	0.01260	
Mucus or bloody stool	H4	-0.01418	0.01418		0.00549	0.00053	
CRC in 1st degree relatives	H4	-0.75691	0.75691		0.29331	0.02806	
Age at screening	H5	0.29125	0.29125	2.23394	0.13037	0.01250	
Diagnosis of any cancer	H5	-0.27995	0.27995		0.12532	0.01202	
Positive FIT (1-sample)	H5	-0.68130	0.68130		0.30498	0.02924	
Chronic diarrhea	H5	-0.26016	0.26016		0.11646	0.01117	
Male	H5	0.58311	0.58311		0.26102	0.02503	
Mucus or bloody stool	H5	0.06495	0.06495		0.02907	0.00279	
CRC in 1st degree relatives	H5	-0.07323	0.07323		0.03278	0.00314	
Age at screening	H6	-0.05070	0.05070	2.04798	0.02476	0.00133	
Diagnosis of any cancer	H6	-0.20665	0.20665		0.10091	0.00543	
Positive FIT (1-sample)	H6	-0.53102	0.53102		0.25929	0.01395	
Chronic diarrhea	H6	-0.16010	0.16010		0.07818	0.00421	
Male	H6	0.57280	0.57280		0.27969	0.01505	
Mucus or bloody stool	H6	0.18476	0.18476		0.09022	0.00485	
CRC in 1st degree relatives	H6	-0.34194	0.34194		0.16697	0.00898	
Age at screening	H7	-0.13947	0.13947	3.44271	0.04051	0.00654	
Diagnosis of any cancer	H7	0.56167	0.56167		0.16315	0.02634	
Positive FIT (1-sample)	H7	1.71461	1.71461		0.49804	0.08041	
Chronic diarrhea	H7	0.33761	0.33761		0.09807	0.01583	

Male	H7	0.24728	0.24728		0.07183	0.01160
Mucus or bloody stool	H7	-0.06743	0.06743		0.01959	0.00316
CRC in 1st degree relatives	H7	0.37464	0.37464		0.10882	0.01757
Age at screening	H8	0.16837	0.16837	1.20702	0.13949	0.00945
Diagnosis of any cancer	H8	0.22406	0.22406		0.18563	0.01258
Positive FIT (1-sample)	H8	0.09137	0.09137		0.07570	0.00513
Chronic diarrhea	H8	-0.16035	0.16035		0.13285	0.00900
Male	H8	0.14951	0.14951		0.12387	0.00839
Mucus or bloody stool	H8	-0.21037	0.21037		0.17429	0.01181
CRC in 1st degree relatives	H8	-0.20300	0.20300		0.16818	0.01139
Age at screening	H9	0.57272	0.57272	4.14829	0.13806	0.02054
Diagnosis of any cancer	H9	0.07103	0.07103		0.01712	0.00255
Positive FIT (1-sample)	H9	-0.68076	0.68076		0.16411	0.02442
Chronic diarrhea	H9	-0.48120	0.48120		0.11600	0.01726
Male	H9	0.17806	0.17806		0.04292	0.00639
Mucus or bloody stool	H9	0.91874	0.91874		0.22147	0.03295
CRC in 1st degree relatives	H9	1.24579	1.24579		0.30031	0.04468
Age at screening	H10	0.02738	0.02738	0.58871	0.04651	0.00231
Diagnosis of any cancer	H10	-0.17758	0.17758		0.30164	0.01501
Positive FIT (1-sample)	H10	0.03778	0.03778		0.06417	0.00319
Chronic diarrhea	H10	0.08230	0.08230		0.13980	0.00696
Male	H10	-0.04666	0.04666		0.07926	0.00394
Mucus or bloody stool	H10	0.20864	0.20864		0.35440	0.01764
CRC in 1st degree relatives	H10	-0.00837	0.00837		0.01422	0.00071
Age at screening	H11	-0.29220	0.29220	3.56899	0.08187	0.00969
Diagnosis of any cancer	H11	-0.54101	0.54101		0.15159	0.01794
Positive FIT (1-sample)	H11	-1.46712	1.46712		0.41107	0.04865
Chronic diarrhea	H11	-0.13045	0.13045		0.03655	0.00433
Male	H11	0.08989	0.08989		0.02519	0.00298
Mucus or bloody stool	H11	0.50190	0.50190		0.14063	0.01664
CRC in 1st degree relatives	H11	-0.54642	0.54642		0.15310	0.01812
H1	CRC	0.25359	0.25359	7.79961	0.03251	
H10	CRC	0.38816	0.38816		0.04977	
H11	CRC	-0.92309	0.92309		0.11835	
H2	CRC	-0.87356	0.87356		0.11200	
H3	CRC	-0.49969	0.49969		0.06407	
H4	CRC	0.74603	0.74603		0.09565	
H5	CRC	0.74781	0.74781		0.09588	
H6	CRC	-0.41957	0.41957		0.05379	
H7	CRC	1.25921	1.25921		0.16144	
H8	CRC	-0.52840	0.52840		0.06775	
H9	CRC	1.16050	1.16050		0.14879	

FIT, faecal immunochemical test; CRC, colorectal cancer. Note: u_{jk} is calculated as the absolute value of weight between predictor variable k and hidden unit j divided by total absolute values of weights pointing to hidden unit j; v_j is calculated as the absolute value of weight between hidden unit j and the outcome divided by total absolute values of weights pointing to the outcome; C_k represents the contribution of predictor variable k on the outcome; the sum of contributions of all predictor variables is 100%.

Supplementary Table 4. Weights from the input variables to the hidden layer neurons, and from the hidden layer neurons to the outcome variable in the artificial neural network model incorporating risk factors and 2-sample FIT results

From	To	Weight	Abs (weight)	Sum	Abs (weight)/Sum	$v_j * u_{jk}$	C_k
Age at screening	H1	0.06226	0.06226	2.58085	0.02412	0.00307	0.076
Diagnosis of any cancer	H1	0.29865	0.29865		0.11572	0.01471	0.118
Positive FIT (2-sample)	H1	0.76278	0.76278		0.29555	0.03756	0.394
Chronic diarrhea	H1	0.39843	0.39843		0.15438	0.01962	0.107
Male	H1	0.06582	0.06582		0.02550	0.00324	0.073
Mucus or bloody stool	H1	-0.51367	0.51367		0.19903	0.02530	0.097
CRC in 1st degree relatives	H1	0.47924	0.47924		0.18569	0.02360	0.135
Age at screening	H2	-0.00613	0.00613	2.00414	0.00306	0.00031	
Diagnosis of any cancer	H2	0.34970	0.34970		0.17449	0.01791	
Positive FIT (2-sample)	H2	0.94350	0.94350		0.47078	0.04832	
Chronic diarrhea	H2	0.10637	0.10637		0.05308	0.00545	
Male	H2	0.24056	0.24056		0.12003	0.01232	
Mucus or bloody stool	H2	0.21208	0.21208		0.10582	0.01086	
CRC in 1st degree relatives	H2	-0.14581	0.14581		0.07275	0.00747	
Age at screening	H3	0.32143	0.32143	3.74331	0.08587	0.01500	
Diagnosis of any cancer	H3	0.62224	0.62224		0.16623	0.02905	
Positive FIT (2-sample)	H3	1.43561	1.43561		0.38351	0.06702	
Chronic diarrhea	H3	0.15308	0.15308		0.04089	0.00715	
Male	H3	0.04457	0.04457		0.01191	0.00208	
Mucus or bloody stool	H3	-0.43135	0.43135		0.11523	0.02014	
CRC in 1st degree relatives	H3	0.73503	0.73503		0.19636	0.03431	
Age at screening	H4	-0.51833	0.51833	2.51825	0.20583	0.02999	
Diagnosis of any cancer	H4	0.14509	0.14509		0.05761	0.00839	
Positive FIT (2-sample)	H4	0.51639	0.51639		0.20506	0.02988	
Chronic diarrhea	H4	0.25205	0.25205		0.10009	0.01458	
Male	H4	-0.23620	0.23620		0.09380	0.01367	
Mucus or bloody stool	H4	-0.31348	0.31348		0.12448	0.01814	
CRC in 1st degree relatives	H4	-0.53671	0.53671		0.21313	0.03106	
Age at screening	H5	0.01739	0.01739	1.94922	0.00892	0.00108	
Diagnosis of any cancer	H5	-0.22914	0.22914		0.11755	0.01423	
Positive FIT (2-sample)	H5	-0.93668	0.93668		0.48054	0.05818	
Chronic diarrhea	H5	-0.21051	0.21051		0.10800	0.01308	
Male	H5	0.15961	0.15961		0.08189	0.00991	
Mucus or bloody stool	H5	0.16404	0.16404		0.08416	0.01019	
CRC in 1st degree relatives	H5	-0.23185	0.23185		0.11894	0.01440	
Age at screening	H6	-0.10081	0.10081	0.51943	0.19408	0.00358	
Diagnosis of any cancer	H6	0.04294	0.04294		0.08267	0.00153	
Positive FIT (2-sample)	H6	-0.20618	0.20618		0.39692	0.00733	
Chronic diarrhea	H6	0.07475	0.07475		0.14390	0.00266	
Male	H6	0.02520	0.02520		0.04851	0.00090	
Mucus or bloody stool	H6	-0.05579	0.05579		0.10741	0.00198	
CRC in 1st degree relatives	H6	-0.01376	0.01376		0.02650	0.00049	
Age at screening	H7	-0.17502	0.17502	1.62899	0.10744	0.00414	
Diagnosis of any cancer	H7	0.11573	0.11573		0.07104	0.00274	
Positive FIT (2-sample)	H7	-0.96751	0.96751		0.59393	0.02290	
Chronic diarrhea	H7	0.02135	0.02135		0.01311	0.00051	

Male	H7	0.09152	0.09152		0.05618	0.00217
Mucus or bloody stool	H7	-0.15310	0.15310		0.09399	0.00362
CRC in 1st degree relatives	H7	-0.10476	0.10476		0.06431	0.00248
Age at screening	H8	0.01362	0.01362	1.34920	0.01009	0.00122
Diagnosis of any cancer	H8	-0.15141	0.15141		0.11222	0.01358
Positive FIT (2-sample)	H8	-0.71084	0.71084		0.52686	0.06377
Chronic diarrhea	H8	-0.36175	0.36175		0.26812	0.03245
Male	H8	-0.04465	0.04465		0.03309	0.00401
Mucus or bloody stool	H8	-0.00392	0.00392		0.00290	0.00035
CRC in 1st degree relatives	H8	-0.06302	0.06302		0.04671	0.00565
Age at screening	H9	-0.00319	0.00319	0.85474	0.00373	0.00032
Diagnosis of any cancer	H9	0.13787	0.13787		0.16130	0.01390
Positive FIT (2-sample)	H9	0.34785	0.34785		0.40697	0.03507
Chronic diarrhea	H9	-0.05757	0.05757		0.06735	0.00580
Male	H9	-0.14917	0.14917		0.17452	0.01504
Mucus or bloody stool	H9	-0.02298	0.02298		0.02688	0.00232
CRC in 1st degree relatives	H9	0.13611	0.13611		0.15924	0.01372
Age at screening	H10	0.14494	0.14494	0.49191	0.29464	0.00576
Diagnosis of any cancer	H10	-0.01503	0.01503		0.03055	0.00060
Positive FIT (2-sample)	H10	0.13106	0.13106		0.26643	0.00521
Chronic diarrhea	H10	-0.08442	0.08442		0.17161	0.00335
Male	H10	0.06217	0.06217		0.12638	0.00247
Mucus or bloody stool	H10	0.03420	0.03420		0.06953	0.00136
CRC in 1st degree relatives	H10	0.02010	0.02010		0.04086	0.00080
Age at screening	H11	0.49770	0.49770	1.85152	0.26881	0.01209
Diagnosis of any cancer	H11	0.06258	0.06258		0.03380	0.00152
Positive FIT (2-sample)	H11	0.76513	0.76513		0.41325	0.01858
Chronic diarrhea	H11	0.08364	0.08364		0.04518	0.00203
Male	H11	0.30338	0.30338		0.16386	0.00737
Mucus or bloody stool	H11	0.10202	0.10202		0.05510	0.00248
CRC in 1st degree relatives	H11	0.03706	0.03706		0.02002	0.00090
H1	CRC	-1.70992	1.70992	13.45352	0.12710	
H10	CRC	-0.26284	0.26284		0.01954	
H11	CRC	-0.60485	0.60485		0.04496	
H2	CRC	1.38097	1.38097		0.10265	
H3	CRC	2.35089	2.35089		0.17474	
H4	CRC	-1.96034	1.96034		0.14571	
H5	CRC	-1.62896	1.62896		0.12108	
H6	CRC	0.24840	0.24840		0.01846	
H7	CRC	-0.51868	0.51868		0.03855	
H8	CRC	-1.62831	1.62831		0.12103	
H9	CRC	-1.15937	1.15937		0.08618	

FIT, faecal immunochemical test; CRC, colorectal cancer. Note: u_{jk} is calculated as the absolute value of weight between predictor variable k and hidden unit j divided by total absolute values of weights pointing to hidden unit j; v_j is calculated as the absolute value of weight between hidden unit j and the outcome divided by total absolute values of weights pointing to the outcome; C_k represents the contribution of predictor variable k on the outcome; the sum of contributions of all predictor variables is 100%.

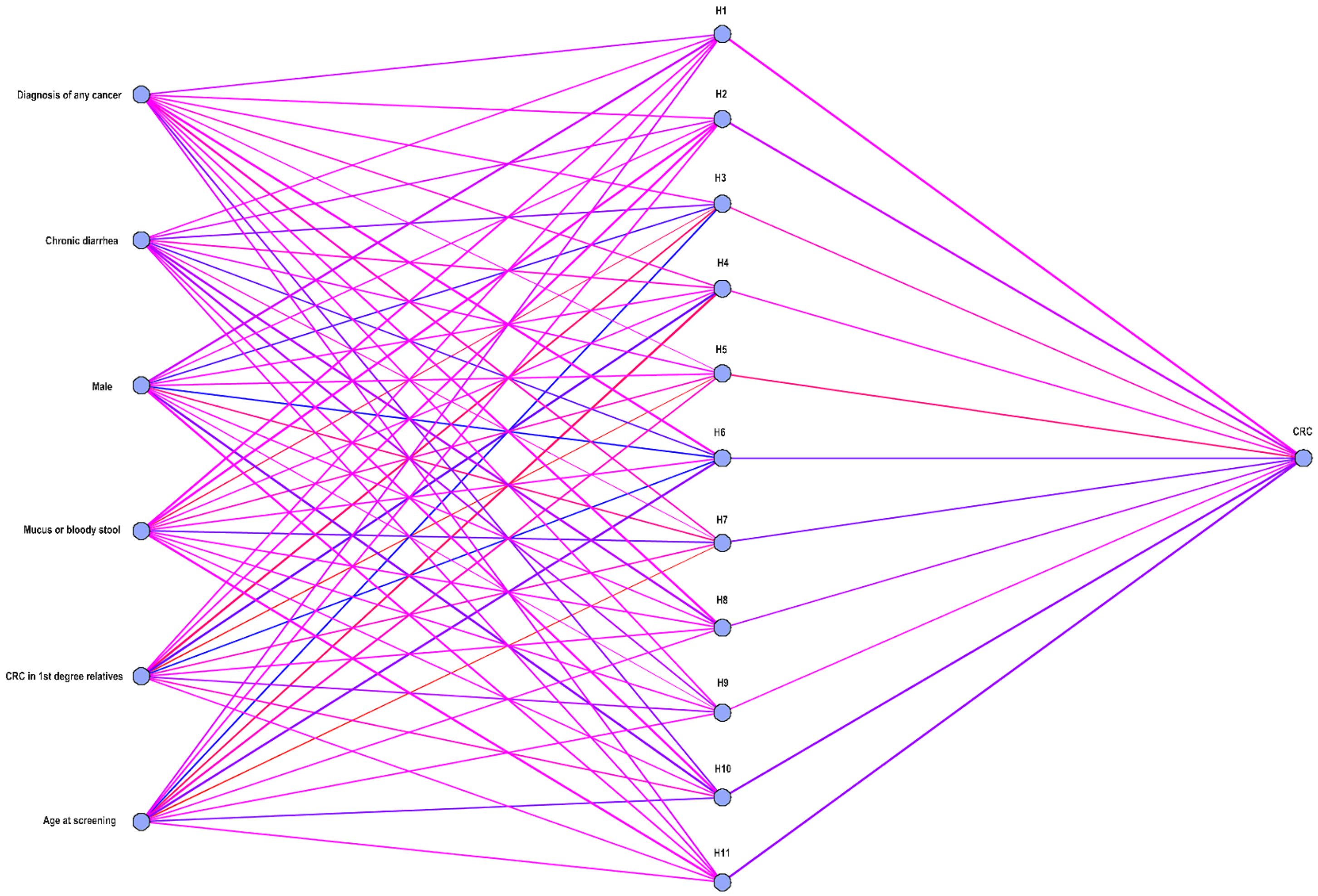

Supplementary Figure 1. Artificial neural network architecture for predicting current risk of CRC using risk factors only.

CRC, colorectal cancer. Note: blue, red and purple connecting lines indicated negative weights, positive weights and small weights, respectively.

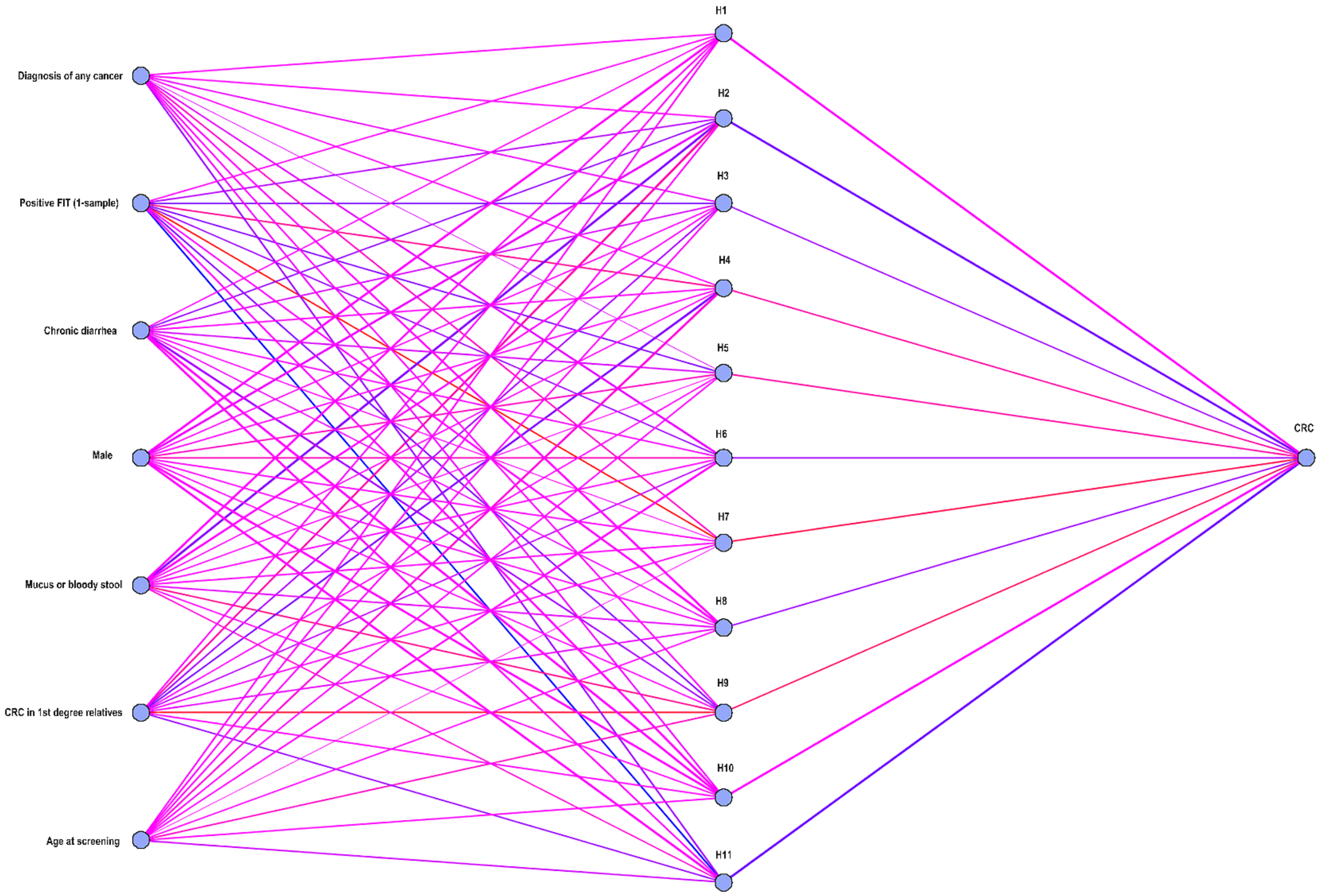

Supplementary Figure 2. Artificial neural network architecture for predicting current risk of CRC using risk factors and 1-sample FIT results.

CRC, colorectal cancer; FIT, faecal immunochemical test. Note: blue, red and purple connecting lines indicated negative weights, positive weights and small weights, respectively.

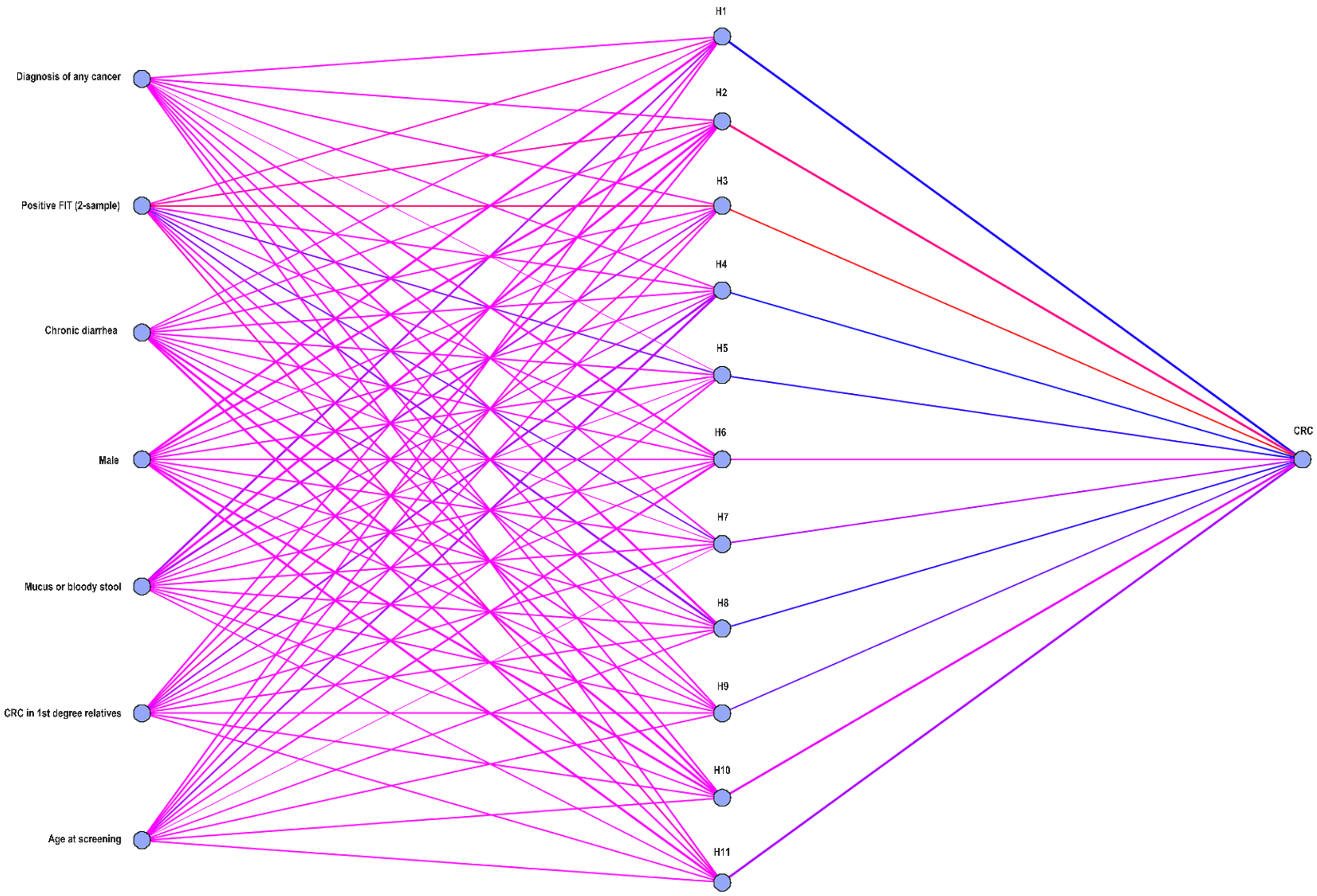

Supplementary Figure 3. Artificial neural network architecture for predicting current risk of CRC using risk factors and 2-sample FIT results.

CRC, colorectal cancer; FIT, faecal immunochemical test. Note: blue, red and purple connecting lines indicated negative weights, positive weights and small weights, respectively.

Risk predictive models	AUC (95% CI)		
	Without FIT	1-sample FIT	2-sample FIT
**Derivation set**			
Support vector machine	0.645 (0.633, 0.658)	0.777 (0.764, 0.789)	0.810 (0.798, 0.821)
Random forest	0.651 (0.638, 0.664)	0.779 (0.767, 0.792)	0.812 (0.801, 0.824)
**Validation set**			
Support vector machine	0.643 (0.627, 0.660)	0.785 (0.770, 0.800)	0.810 (0.796, 0.824)
Random forest	0.644 (0.628, 0.660)	0.782 (0.767, 0.797)	0.811 (0.797, 0.825)

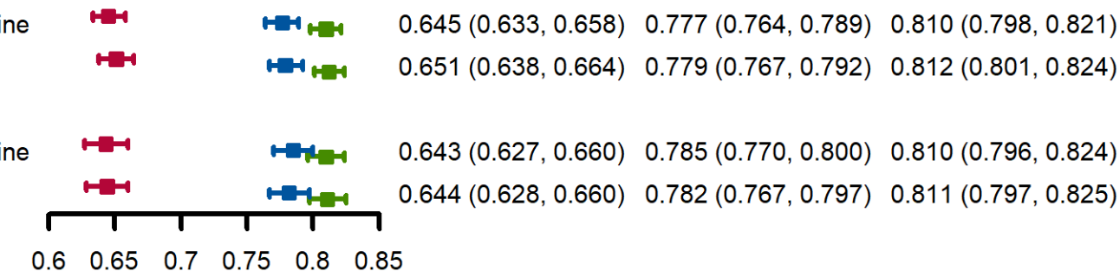

Supplementary Figure 4. Area under the receiver operating characteristic curve (AUC) of SVM and RF models for colorectal cancer in the derivation and validation sets.

CI, confidence interval; SVM, support vector machine; RF, random forest.

	AUC (95% CI)		
	**Without FIT**	**1-sample FIT**	**2-sample FIT**
**Derivation set (1-year interval)**			
**Risk predictive models**			
Logistic regression	0.655 (0.640, 0.670)	0.806 (0.792, 0.820)	0.850 (0.838, 0.861)
Artificial neural network	0.658 (0.643, 0.673)	0.807 (0.793, 0.821)	0.849 (0.837, 0.860)
**Risk scoring systems**			
LR-based	0.650 (0.635, 0.665)	0.804 (0.790, 0.819)	0.845 (0.833, 0.857)
ANN-based	0.648 (0.633, 0.663)	0.803 (0.789, 0.817)	0.845 (0.833, 0.857)
**Validation set (1-year interval)**			
**Risk predictive models**			
Logistic regression	0.661 (0.642, 0.680)	0.838 (0.823, 0.854)	0.868 (0.855, 0.881)
Artificial neural network	0.664 (0.646, 0.683)	0.838 (0.822, 0.853)	0.873 (0.861, 0.886)
**Risk scoring systems**			
LR-based	0.652 (0.634, 0.671)	0.835 (0.820, 0.851)	0.871 (0.859, 0.884)
ANN-based	0.650 (0.631, 0.669)	0.832 (0.816, 0.848)	0.870 (0.857, 0.883)
**Derivation set (1.5-year interval)**			
**Risk predictive models**			
Logistic regression	0.651 (0.637, 0.665)	0.798 (0.785, 0.811)	0.831 (0.819, 0.842)
Artificial neural network	0.652 (0.639, 0.666)	0.799 (0.786, 0.812)	0.832 (0.821, 0.843)
**Risk scoring systems**			
LR-based	0.646 (0.632, 0.660)	0.787 (0.773, 0.800)	0.822 (0.810, 0.833)
ANN-based	0.642 (0.628, 0.656)	0.783 (0.770, 0.796)	0.820 (0.808, 0.832)
**Validation set (1.5-year interval)**			
**Risk predictive models**			
Logistic regression	0.655 (0.638, 0.672)	0.798 (0.781, 0.814)	0.833 (0.818, 0.847)
Artificial neural network	0.655 (0.638, 0.672)	0.799 (0.782, 0.815)	0.835 (0.821, 0.849)
**Risk scoring systems**			
LR-based	0.649 (0.633, 0.666)	0.811 (0.796, 0.827)	0.846 (0.832, 0.859)
ANN-based	0.649 (0.632, 0.666)	0.806 (0.790, 0.822)	0.843 (0.829, 0.857)

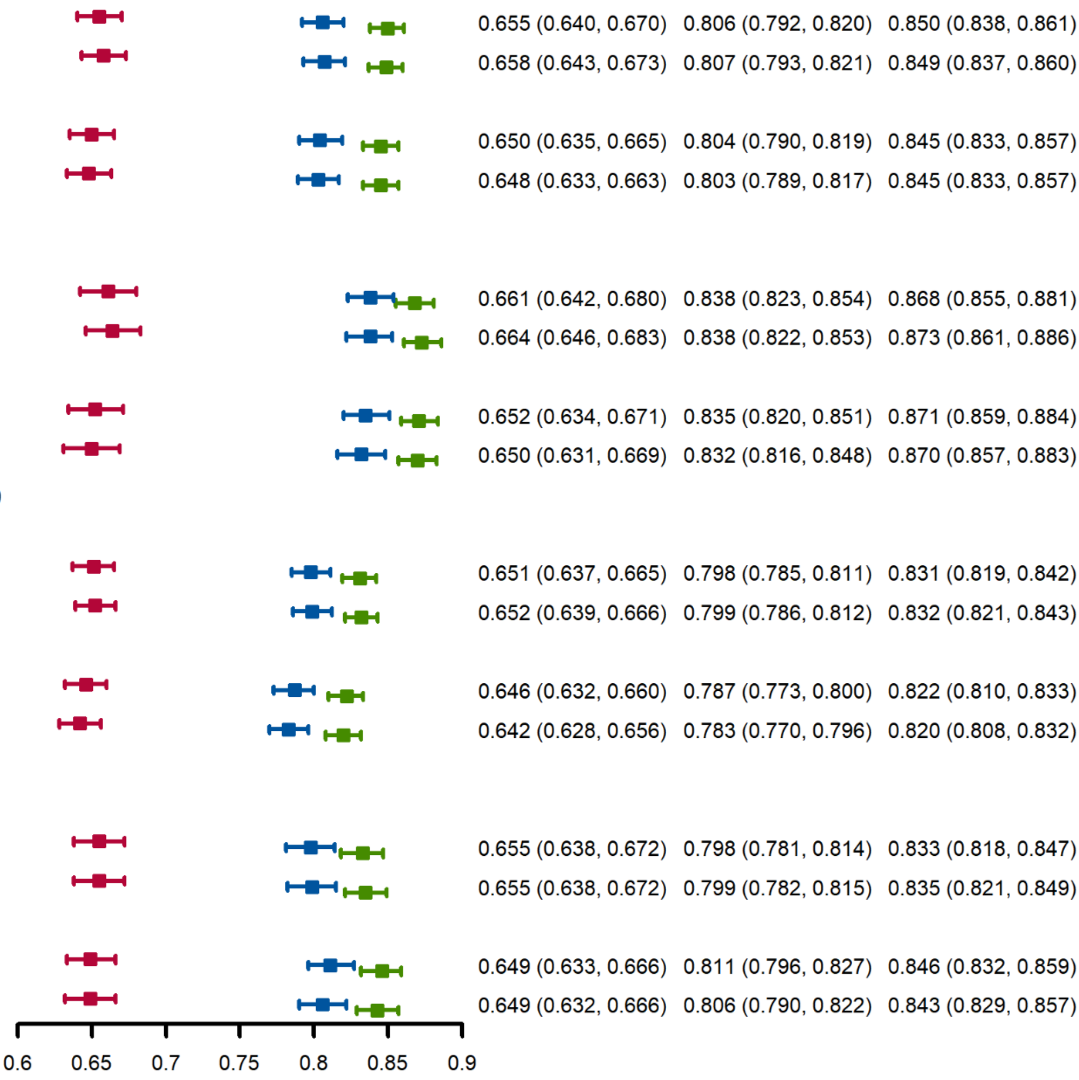

Supplementary Figure 5. Sensitivity analysis for area under the receiver operating characteristic curve (AUC) of predictive models and scoring systems for colorectal cancer in the derivation and validation sets.

CI, confidence interval; LR, logistic regression; ANN, artificial neural network.
